# The ^15^N-leucine single-injection method allows for determining endogenous losses and true digestibility of amino acids in cecectomized roosters

**DOI:** 10.1371/journal.pone.0188525

**Published:** 2017-11-22

**Authors:** Rujiu Hu, Jing Li, Rab Nawaz Soomro, Fei Wang, Yan Feng, Xiaojun Yang, Junhu Yao

**Affiliations:** 1 College of Animal Science and Technology, Northwest A&F University, Yangling, Shaanxi, China; 2 College of Life Science, Shanxi Agriculture University, Jinzhong, Shanxi, China; Leibniz-Institut fur Pflanzengenetik und Kulturpflanzenforschung Gatersleben, GERMANY

## Abstract

This study was conducted to assess the influence of dietary protein content in poultry when using the ^15^N-leucine single-injection method to determine endogenous amino acid losses (EAALs) in poultry. Forty-eight cecectomized roosters (2.39 ± 0.23 kg) were randomly allocated to eight dietary treatments containing protein levels of 0, 3%, 6%, 9%, 12%, 15%, 18% and 21%. Each bird was precisely fed an experimental diet of 25 g/kg of body weight. After feeding, all roosters were subcutaneously injected with a ^15^N-leucine solution at a dose of 20 mg/kg of body weight. Blood was sampled 23 h after the injection, and excreta samples were continuously collected during the course of the 48-h experiment. The ratio of ^15^N-enrichment of leucine in crude mucin to free leucine in plasma ranged from 0.664 to 0.763 and remained relatively consistent (*P* > 0.05) across all treatments. The amino acid (AA) profiles of total endogenous AAs, except isoleucine, alanine, aspartic acid, cysteine, proline and serine, were not influenced (*P* > 0.05) by dietary protein contents. The predominant endogenous AAs in the excreta were glutamic acid, aspartic acid, threonine, serine and proline. The order of the relative proportions of these predominant AAs also remained relatively constant (*P* > 0.05). The endogenous losses of total AAs determined with the ^15^N-leucine single-injection method increased curvilinearly with the dietary protein contents. The true digestibility of most AAs and total AAs was independent of their respective dietary protein levels. Collectively, the ^15^N-leucine single-injection method is appropriate for determining EAALs and the true digestibility of AAs in poultry fed varying levels of protein-containing ingredients.

## Introduction

The accurate quantification of endogenous amino acids losses (EAALs) in the intestines of animals is crucial for determining amino acid (AA) requirements and for calculating the true AA digestibility of feedstuffs. In poultry, EAALs have traditionally been determined by the fasted cecectomized rooster method, linear regression and the nitrogen-free diet method [1]. However, these methods can only be used to determine basal (or diet-independent) EAALs. Some dietary ingredients associated with “diet-specific” losses induce higher than basal losses [2]. To determine the total EAALs (basal + specific) in poultry fed various types of diets, our group proposed a method with a single injection of ^15^N-leucine (i.e., the ^15^N-leucine single-injection method) [3], which allowed us to estimate the true AA digestibility of feedstuffs. Compared with the traditional ^15^N-isotope infusion method that involves the continuous infusion of a^15^N-labeled AA [4–6], our method has advantages such as low cost and simplicity. This method mainly relies on the assumption that the ratio of ^15^N-enrichment of endogenous leucine in excreta (NL_*e*_) to leucine in deproteinized plasma (NL_*p*_) remains relatively constant (i.e., the NL_*e*_/NL_*p*_ ratio is a constant value) after the injection of a single bolus of ^15^N-leucine into birds fed different protein-containing diets. Our previous results supported this method with dietary crude protein (CP) levels ranging from 0 to 5% [3]. However, further data are needed to assess the influence of higher dietary CP levels before practical application. Because the value of NL_*e*_ cannot be determined directly due to interference from undigested dietary leucine, obtaining an alternative technique that reflects the value of NL_*e*_ is the key to evaluating the method.

The gut mucosa is the biggest source of EAALs in the intestinal lumen [5, 7, 8]. Moreover, findings by Selle et al. [9] and Miner-Williams et al. [10] indicated that the mucin content was highest in all components of endogenous protein in the digesta. Mucin protein accounted for more than half of the non-bacterial protein components in the digesta [11]. Based on the above findings, we assume that mucin excretion can be taken individually as a reference for the whole endogenous protein content in the excreta. Therefore, we can investigate the effect of dietary CP levels on the NL_*cm*_ (^15^N-enrichment of leucin in crude mucin)/NL_*p*_ ratio to evaluate the ^15^N-leucine single-injection method.

Consistent with the ^15^N-isotope dilution method, the ^15^N-leucine single-injection method can only be used to estimate the endogenous losses of the labeled AA and not the levels of each AA [12]. A relatively constant AA profile of total endogenous protein is assumed to calculate the endogenous loss of each AA [4, 13], but few data are available in support of this hypothesis. In the present study, we proposed a gradient protein method to obtain the AA profile of total endogenous AAs in poultry fed protein-containing diets. The gradient protein method depends on the theory that the true AA digestibility is independent of the dietary CP level when the ingredient composition of the diet is constant [2, 14, 15].

The objectives of this study were: 1) to investigate the effect of dietary CP levels on the NL_*cm*_/NL_*p*_ ratio using crude mucin in excreta as a reference for the whole endogenous protein in the excreta and 2) to obtain the AA profiles of total endogenous AAs using the gradient protein method and 3) to obtain estimates of the true digestibility of AAs in cecectomized roosters.

## Materials and methods

### Ethics statement

The experimental proposal and surgical procedures were approved by the Northwest A&F University Animal Care and Use Committee.

### Animals and diets

The animal feeding experiment was performed at the Experimental Center of Animal Science at Northwest A&F University. A total of 48 cecectomized Lohmann Brown roosters (35 wks old), with an average body weight of 2.39 ± 0.23 kg, were obtained from the Da Cheng Poultry Industry (Xianyang, Shaanxi Province, China). Cecectomy and postoperative care were performed as described by Payne et al. [16]. All birds were individually raised in a single cage under a 16 h light and 8 h dark photoperiod. Drinking water was offered *ad libitum*. The birds were randomly divided among 8 dietary treatments. The experimental diets included a nitrogen-free diet and seven protein-containing diets (Table 1). Soybean meal was the only source of dietary CP. The AA composition of the diets is shown in S1 Table. All other nutrients and energy met or exceeded the estimated requirements for roosters [17]. A fiber source was used to equalize the crude fiber levels in all experimental diets. The electrolyte balance was constant across all the diets recommended by Adedokun et al. [1].

**Table 1:** Ingredient composition of the experimental diets (g/kg as-fed basis).

Item	Dietary CP level (%)								Pooled SEM	*P*-value
	0	3	6	9	12	15	18	21		
Ingredient										
Corn starch	650.4	587.7	523.9	462.2	401.4	335.1	275.5	215.4	-	-
Soybean meal	0.0	69.0	138.0	207.0	273.0	345.0	414.0	483.0	-	-
Sucrose	210.0	215.0	220.0	225.0	230.0	235.0	238.0	240.0	-	-
Cellulose	60.0	51.2	43.7	33.9	26.3	18.3	8.5	0.0	-	-
Soybean oil	20.0	20.0	20.0	20.0	20.0	20.0	20.0	20.0	-	-
Premix^1^	10.0	10.0	10.0	10.0	10.0	10.0	10.0	10.0	-	-
CaHPO_4_	19.0	18.5	17.9	17.4	16.8	16.3	15.7	15.3	-	-
CaCO_3_	10.0	9.8	9.6	9.4	9.2	9.0	8.8	8.6	-	-
K_2_CO_3_^2^	11.7	10.1	8.4	6.7	5.1	3.3	1.6	0.0	-	-
KCl^2^	2.2	2.1	2.0	1.9	1.9	1.8	1.7	1.6	-	-
NaHCO_3_^2^	3.7	3.6	3.5	3.4	3.4	3.3	3.2	3.1	-	-
Choline chloride	3.0	3.0	3.0	3.0	3.0	3.0	3.0	3.0	-	-
Chemical composition^3^										
ME (kJ/kg)	2969	2965	2958	2957	2952	2945	2943	2936	2.3	0.05
CP	2.2^h^	32.4^g^	62.7^f^	92.9^e^	121.9^d^	153.4^c^	183.7^b^	213.9^a^	11.1	<0.001
CF	29.4	29.1	29.5	28.8	29	29.3	28.6	28.5	0.27	0.971
NDF	55.2	54.5	55.5	54.4	55.1	55.9	54.9	55.1	0.33	0.963
Ca	9.5	9.6	9.4	9.4	9.4	9.5	9.4	9.4	0.09	0.999
Available P	4.3	4.2	4.4	4.6	4.4	4.3	4.4	4.5	0.09	0.967

CP = crude protein; CF = crude fiber; ME = metabolizable energy; NDF = neutral-detergent fiber.

^1^Supplied (per kg of diet): vitamin A 5000 IU, vitamin D_3_ 1500 IU, vitamin E 30 IU, vitamin K 3 mg, vitamin B_1_ 4 mg, vitamin B_2_ 6 mg, calcium pantothenate 15 mg, niacin 35 mg, vitamin B_6_ 4 mg, folic acid 5 mg, cyanocobalamin 30 μg, biotin 0.3 mg, iron 60 mg, copper 10 mg, manganese 80 mg, zinc oxide 80 mg, selenium 0.3 mg, potassium iodate 0.9 mg, antioxidant 100 mg.

^2^Na^+^ + K^+^ − Cl^−^ − milliequivalent value of 203.

^3^Values are means with pooled SEM (n = 5). The degrees of freedom between groups are 7 and those within groups are 32. The F-ratios for ME, CP, CF, NDF, Ca and available P are 3.704, 3611.58, 0.243, 0.264, 0.068 and 0.253, respectively.

^a-h^Means with the same superscript letters in the row are not significantly different (*P* > 0.05), and those with different letters are significantly different (*P* < 0.05).

### ^15^N-Leucine injection and sampling

The general outline of the ^15^N-leucine injection and sampling protocol is summarized in S2 Table. The experiment included three periods. In the first period, the birds were adapted to the experimental diets for 5 consecutive days. On day 6, the roosters were deprived of the diet for 24 h to empty the digestive tract of all dietary residues. Then, the birds were precision-fed 25.0 g of dry matter/kg of body weight of each experimental diet using the method presented by Kim et al. [18]. Following the precision feeding, excreta were collected quantitatively for 48 h. A bag (250 mL) was attached to a hollowed-out plastic bottle cap fitted to the rooster’s cloaca. Then, 10 mL of a 10% formic acid solution was added to the bags to inhibit enzyme and microbial activity in the excreta. The excreta in the bag was collected once every two hours, and frozen (−40°C) immediately. The 48-h excreta samples were pooled for each bird when the experiment was over. Blood samples (5 mL) were sampled 23 h after precision feeding [3]. The above blood and excreta samples were used to determine the basal ^15^N-enrichment of leucine in the birds. In the second period, all birds were given one week to recover from the first period. In the third period, all roosters were subcutaneously injected with ^15^N-L-leucine (98% ^15^N-enrichment; Cambridge Isotope Labs. Inc., Andover, MA, USA) at a dose of 20 mg/kg of body weight after precision feeding. The other procedures were the same as those described for the first period.

### Sample preparation

The sample preparation protocol was adapted from the study by Steendam et al. [19]. Briefly, blood samples were centrifuged (10 min, 2000×g, 4°C) to recover plasma and then treated twice with a 10% (W/V) trichloroacetic acid solution to obtain deproteinized plasma. The AAs in the deproteinized plasma were purified though passage on AG 50W-X8 cation-exchange resin (hydrogen form, 200 μm, Sigma, St. Louis, MO, USA). The AAs were eluted with 4 mol/L fresh NH_4_OH. After NH_4_OH evaporation, the samples were re-dissolved with 5 mL of ultrapure water for the ^15^N analysis. The excreta samples were thawed, homogenized, freeze-dried, weighed and then carefully ground to pass through a 1 mm screen for further analysis. Crude mucin in the excreta was isolated with the ethanol precipitation method described by Lien et al. [5] and Leterme et al. [20]. Hydrolyzed excreta or crude mucin samples that were obtained using hydrochloric acid method (10 mL of 6 mol/L HCl, 110°C, 24 h) were purified similarly to the plasma samples for ^15^N analysis.

### Chemical and isotope analyses

Dry matter, CP (N × 6.25) and neutral detergent fiber were analyzed in the ingredients and diets [21]. The AA concentrations of the ingredients, diets and excreta samples were analyzed using the Biochrom 30 amino acid analyzer (Pharmacia Biotech, Cambridge, UK). The ^15^N-enrichment of leucine was determined with a gas chromatograph coupled with a combustion oven and an isotope ratio mass spectrometer (GC-C-IRMS, Thermo-Finngan, USA) system according to a previously presented study [4]. The AAs were derivatized with thionyl chloride and pivaloyl chloride [22], which led to the formation of N-pivaloyl-isopropyl derivatives. The samples (1.0 μL, split mode = 10:1) were injected onto an HP ULTRA 2 column (50 m × 0.32 mm × 0.52 μm) at an injector temperature of 250°C. The column oven was initially set at 70°C (for 1 min), rise at a rate of 3°C/min to 220°C, and then 10°C/min to 300°C with a holding time of 8 min. The temperatures of the CuO/Pt combustion reactor and reduction oven were 850°C and 650°C, respectively.

### Principles of calculations

#### ^15^N-enrichment in leucine

The ^15^N-enrichment (atom percent excess, APE) in leucine was obtained according to formulas (1) and (2), which were adapted from Wolfe et al. [23]:

$$APE(\%)=\frac{TTR}{TTR+1}\times 100\%$$ (1)

$$TTR(\%)=[{\left(15N/14N\right)}_{sam}-{\left(15N/14N\right)}_{bas}]\times (1-A)$$ (2)

where the 15N/14N ratios originate from the ratios of the m/z 29 to m/z 28 ion current signals in a labeled sample (sam) or in basal sample (bas) [22]. The value of A is the natural abundance of ^15^N.

#### The ^15^N-leucine single-injection method

The calculation principle of this method, which has been shown previously [3], is expressed as the formula (3):

$$\frac{{NL}_{e0}}{{NL}_{p0}}=\frac{{NL}_{ei}}{{NL}_{pi}}$$ (3)

where *i* denotes the number of experimental protein-containing diets, and NL_*e0*_/NL_*p0*_ and NL_*ei*_/NL_*pi*_ are the ^15^N-enrichment ratios of endogenous leucine in the excreta and free leucine in the deproteinized plasma from the birds fed either nitrogen-free diet or the *i*^th^ protein-containing diet, respectively.

The ^15^N-leucine content in the excreta was derived from endogenous ^15^N-leucine; therefore, we obtained formula (4). By combining formulas (3) and (4), we generated the formula (5):

$$W_{ei}\times {NL}_{ei}=W_{ti}\times {NL}_{exi}$$ (4)

$$W_{ei}=\frac{{NL}_{exi}\times {NL}_{p0}}{{NL}_{pi}\times {NL}_{e0}}\times W_{ti}$$ (5)

where NL_*ex*_ represents ^15^N-enrichment of total leucine in the excreta; W_*e*_ and W_*t*_ (mg/kg of dry matter intake (DMI)) are the losses of endogenous leucine and total leucine in the excreta, respectively.

The AA profile of total endogenous AAs was assumed to be relatively stable [4, 13]. Therefore, the AA profile obtained from the nitrogen-free diet could be used to calculate the endogenous losses of other AAs in birds fed protein-containing diets.

#### The gradient protein method for estimating the total endogenous AAs profile

Apparent digestibility (AD) and true digestibility (TD) of AAs in diets were calculated according to formulas (6) and (7):

$${AD}_{i}=\left(1-\frac{{TF}_{i}}{TIi}\right)\times 100\%$$ (6)

$${TD}_{i}={AD}_{i}+\frac{E_{i}}{{TI}_{i}}\times 100\%$$ (7)

where TI, TF and E denote the total AA input (mg/kg of DMI), total fecal AA output (mg/kg of DMI) and EAALs (mg/kg of DMI) from the experimental diets, respectively.

When the source of dietary protein and the contents of the other dietary components, including fiber and anti-nutritive factors, are similar among the experimental diets, then the TD will be the same at varying dietary CP levels [2, 15]. Therefore,

$${TD}_{i+1}={TD}_{i}$$ (8)

Based on several previous studies [24–26], we assumed that the dietary CP levels, which varied within a small range (3%), had little effect on the EAALs.

$$E_{i+1}\approx E_{i}$$ (9)

According to formulas (6), (7), (8) and (9), we obtained formula (10):

$${\bar{E}}_{i(i+1)}\approx \frac{{TI}_{i+1}\times {TF}_{i}-{TI}_{i}\times {TF}_{i+1}}{{TI}_{i+1}-{TI}_{i}}$$ (10)

where ${\bar{E}}_{i(i+1)}$ (mg/kg of DMI) represents the mean endogenous losses of AAs between the *i*^th^ and (*i*+1)^th^ protein-containing diets.

The profile of the total endogenous AAs, expressed as a proportion of total AAs, was calculated as follows:

$${\bar{P}}_{i(i+1)}(\%)\approx \frac{{\bar{E}}_{i(i+1)}}{{\bar{TE}}_{i(i+1)}}\times 100\%$$ (11)

where ${\bar{P}}_{i(i+1)}$ and ${\bar{TE}}_{i(i+1)}$ (mg/kg of DMI) represented mean proportion of each individual AA and the total endogenous losses of AAs between the *i*^th^ and (*i*+1)^th^ protein-containing diets, respectively.

### Statistical analysis

Statistical analysis was performed using SPSS 21.0 (IBM-SPSS Inc., Chicago, Il, USA). The data are presented as the means and pooled standard error (SEM) (n = 6). Differences between the means of all groups were compared by one-way ANOVA, followed by Bonferroni corrections test. Significant differences were declared at *P*<0.05.

## Results

The values of ^15^N-enrichment of leucine in the deproteinized plasma, crude mucin and excreta are presented in Table 2. The values of ^15^N-enrichment were significantly different in the different samples for all treatments. The highest ^15^N-enrichment was observed in the deproteinized plasma, followed by the crude mucin and then the excreta. All the values of ^15^N-enrichment significantly decreased (*P* < 0.05) when the dietary CP level was increased to 15%. The ratios of ^15^N-enrichment in the different samples are also shown in Table 2. The value for NL_*cm*_/NL_*p*_ ratios ranged from 0.664 to 0.763, whereas the NL_*ex*_/NL_*p*_ ratios ranged from 0.262 to 0.744. The dietary CP levels had no influence (*P* > 0.05) on the NL_*cm*_/NL_*p*_ ratios.

**Table 2:** ^15^N-enrichment of leucine in the deproteinized plasma, crude mucin and excreta sampled from precision-fed cecectomized roosters after a single-injection of ^15^N-leucine^1^.

Item	Dietary CP level (%)								Pooled SEM	*P*-value
	0	3	6	9	12	15	18	21		
	^15^N-enrichment of leucine (atom % excess, APE)									
Plasma^2^ (NL_*p*_)	0.256^a^	0.254^a^	0.233^a^^b^	0.209^a^^b^	0.182^a^^b^	0.165^b^	0.147^b^	0.135^b^	0.008	<0.001
Crude mucin (NL_*cm*_)	0.183^a^	0.176^a^	0.158^a^^b^	0.147^a^^b^	0.131^a^^b^	0.122^b^	0.110^b^	0.091^b^	0.006	<0.001
Excreta^3^ (NL_*ex*_)	0.188^a^	0.169^a^^b^	0.144^a^^b^	0.116^b^	0.092^b^^c^	0.076^b^^c^	0.050^c^	0.034^c^	0.008	<0.001
	Ratio of ^15^N-enrichment of leucine in different samples									
NL_*cm*_/NL_*p*_	0.720	0.698	0.682	0.702	0.728	0.705	0.763	0.664	0.014	0.778
NL_*ex*_/NL_*p*_	0.744^a^	0.670^a^^b^	0.613^a^^b^	0.559^b^	0.496^b^	0.427^b^^c^	0.338^c^	0.262^c^	0.025	<0.001

CP = crude protein; NL_*p*_ = ^15^N-enrichment of leucine in the deproteinized plasma; NL_*cm*_ = ^15^N-enrichment of leucine in crude mucin; NL_*ex*_ = ^15^N-enrichment of total leucine in excreta; NL_*cm*_/NL_*p*_ = the ratio of NL_*cm*_ to NL_*p*_; NL_*ex*_/NL_*p*_ = the ratio of NL_*ex*_ to NL_*p*_.

^1^Values are means with pooled SEM (n = 6). The degrees of freedom between groups are 7 and those within groups are 40. The F-ratios for NL_*p*_, NL_*cm*_, NL_*ex*_, NL_*cm*_/NL_*p*_ and NL_*ex*_/NL_*p*_ are 9.011, 8.490, 30.043, 0.567 and 30.570, respectively.

^2^Plasma samples were sampled 23.0 h after subcutaneous single-injection of ^15^N-leucine in precision-fed cecectomized roosters; the NL_*p*_ at this time point represents the weighted mean of the definite integral of ^15^N-enrichment in leucine during the course of the 48.0-h experiment [3].

^3^Excreta samples were continuously sampled during the course of the 48.0-h experiment after the precision-feeding and pooled at the end of the experiment. NL_*ex*_ represents the weighted mean of ^15^N-enrichment in pooled excreta samples (Xu et al. 2011).

^a, b, c^Means with the same superscript letters in the row are not significantly different (*P* > 0.05), and those with different letters are significantly different (*P* < 0.05).

The contribution of endogenous leucine to total leucine and the endogenous losses of leucine in the excreta are presented in Table 3. No significant differences (*P* > 0.05) were found between contributions calculated with NL_*e0*_/NL_*p0*_ and NL_*cm*_/NL_*p*_. Endogenous leucine losses calculated with NL_*e0*_/NL_*p0*_ increased significantly (*P* < 0.05) when the dietary CP level increased to 12%.

**Table 3:** Contribution of endogenous leucine to total leucine and endogenous losses of leucine in excreta sampled from precision-fed cecectomized roosters^1^.

Item	Dietary CP level (%)								
	3	6	9	12	15	18	21		
	Contribution (%) of endogenous leucine to total leucine								
Contribution_*e0/p0*_	90.4	83.5	75.4	68.3	58.3	46.6	35.8		
Contribution_*cm/p*_	95.2	90.9	81.2	69.1	61.5	45.5	39.3		
Pooled SEM	2.72	3.72	3.33	3.18	2.38	3.10	2.92		
*P*-value	0.269	0.117	0.089	0.833	0.506	0.805	0.553		
	Total excretion or endogenous losses of leucine (mg/kg of DMI)							Pooled SEM	*P*-value
Total leucine excretion	590.6^d^	784.1^c^^d^	1136.3^c^^d^	1492.8^c^	1688.7^b^	2295.3^b^^c^	3073.6^a^	135.3	<0.001
Endogenous leucine losses^2^	533.4^b^	654.5^b^	856.9^a^	1019.6^a^	984.5^a^^b^	1069.6^a^	1100.4^a^	36.8	<0.001

CP = crude protein; DMI = dry matter intake; Contribution_*e0/p0*_ = contribution (%) of endogenous leucine to total leucine calculated with NL_*e0*_/NL_*p0*_ (NL_*e0*_ = ^15^N-enrichments of leucine in excreta for nitrogen-free diet, NL_*p0*_ = ^15^N-enrichments of leucine in deproteinized plasma for nitrogen-free diet); Contribution_*cm/p*_ = contribution (%) of endogenous leucine to total leucine calculated with NL_*cm*_/NL_*p*_ (NL_*cm*_ = ^15^N-enrichments of leucine in crude mucin for protein-containing diets, NL_*p*_ = ^15^N-enrichments of leucine in deproteinized plasma for protein-containing diets).

^1^Values are means with pooled SEM (n = 6). The degrees of freedom between groups of Contribution_*e0/p0*_ or Contribution_*cm/p*_ are 1 and those within groups are 10. The degrees of freedom between groups of total excretion or endogenous losses of leucine are 6 and those within groups are 35. The F-ratios for 3%, 6%, 9%, 12%, 15%, 18% and 21% CP are 1.370, 2.934, 3.557, 0.047, 0.476, 0.064 and 0.376, respectively. The F-ratios for total excretion and endogenous losses of leucine are 41.476 and 9.780, respectively.

^2^Determined with the ^15^N-leucine single-injection method when Contribution_*e0/p0*_ was used.

^a, b, c, d^Means with the same superscript letters in the row are not significantly different (*P*>0.05), and those with different letters are significantly different (*P*<0.05).

The AA profile of the total endogenous AAs (expressed as a proportion of total endogenous AAs) determined with the gradient protein method is shown in Table 4. The ratios of most of the indispensable AAs (except isoleucine) and glutamic acid were not influenced (*P* > 0.05) by the dietary CP levels. The proportions of isoleucine, aspartic acid and serine in the total endogenous AAs were greater (*P* < 0.05) in diets with increasing CP levels from 12% to 21% than those in the nitrogen-free diet, whereas the proportions of alanine, cysteine and proline were lower (*P* < 0.05). No significant differences (*P* > 0.05) were observed in the ratios of most AAs in the total endogenous AAs when the dietary CP levels were increased from 3% to 12% or from 12% to 21%. The order of the relative proportions of these predominant AAs was similar across all dietary treatments.

**Table 4:** Amino acid profile (%) of total endogenous amino acids calculated with the gradient protein method in precision-fed cecectomized roosters fed the nitrogen-free diet and soybean meal diets at varying crude protein ranges^1^.

Item	Dietary CP level (%)							Pooled SEM	*P-*Value
	0	3~6	6~9	9~12	12~15	15~18	18~21		
Indispensable amino acids									
Arginine	5.40	4.87	4.76	5.04	4.53	3.96	4.31	0.15	0.175
Histidine	4.89	4.26	3.91	3.54	3.59	4.04	4.05	0.18	0.503
Isoleucine	3.07^b^	4.13^a^^b^	4.16^a^^b^	4.19^a^^b^	4.63^a^^b^	4.67^a^^b^	4.81^a^	0.15	0.035
Leucine	4.95	5.35	5.58	5.16	5.22	4.71	4.93	0.14	0.727
Lysine	6.08	5.85	5.54	5.82	5.84	5.00	4.76	0.19	0.438
Methionine	1.01	1.50	1.59	1.78	1.53	1.47	1.57	0.07	0.178
Phenylalanine	4.30	3.90	4.97	5.01	4.89	3.93	3.94	0.16	0.180
Threonine	8.46	8.30	8.22	8.27	8.18	9.08	9.24	0.20	0.711
Valine	6.81	7.18	7.48	7.08	6.74	7.03	7.36	0.21	0.969
Dispensable amino acids									
Alanine	6.24^a^^b^	6.36^a^	5.58^a^	4.58^b^	4.62^a^	5.23^a^	4.25^b^	0.17	<0.001
Aspartic acid	10.50^b^	10.77^b^	10.94^b^	12.59^a^^b^	13.83^a^	14.38^a^	14.62^a^	0.32	<0.001
Cysteine	5.97^a^	5.64^a^	5.24^a^	4.71^a^^b^	4.02^b^	3.97^b^	4.06^b^	0.21	0.021
Glutamic acid	14.42	14.39	14.68	15.17	15.12	15.49	15.54	0.33	0.948
Proline	11.79^a^	10.19^a^^b^	9.31^a^^b^	8.74^a^^b^	8.50^b^	7.05^b^	6.43^b^	0.36	<0.001
Serine	7.12^b^	7.32^b^	8.04^a^^b^	8.34^a^^b^	9.31^a^^b^	10.51^a^	10.13^a^	0.32	0.009

CP = crude protein.

^1^Values are means with pooled SEM (n = 6). The degrees of freedom between groups are 6 and those within groups are 35. The F-ratios are 1.596, 2.896, 1.004, 0.623, 1.605, 2.595, 0.602, 0.216, 0.905, 1.588, 3.410, 6.652, 5.585, 10.664 and 0.268 according to the order of amino acids in the table, respectively.

^a, b^Means with the same superscript letters in the row are not significantly different (*P* > 0.05), and those with different letters are significantly different (*P* < 0.05).

The endogenous losses of other AAs calculated with the AA profile of total endogenous AAs in the nitrogen-free diet are presented in Table 5. The dietary CP levels had a significant effect (*P* < 0.05) on the endogenous loss of most AAs (except histidine). The endogenous loss of total AAs in roosters fed the 21% CP diet was approximately 2.2 times higher than the endogenous losses determined with birds fed the nitrogen-free diet. Similar endogenous losses (*P* > 0.05) of all individual AAs were observed when increasing the dietary CP levels from 0 to 6%. The endogenous losses of most individual AAs and the total AAs increased slightly (*P* > 0.05) when the dietary CP levels were increased from 0 to 6% or from 12% to 21%. The endogenous losses of most individual AAs and the total AAs increased dramatically when the dietary CP levels were increased from 6% to 12% (especially from 6% to 9%).

**Table 5:** Endogenous amino acid losses (mg/kg of DMI) determined with the ^15^N-leucine single-injection method in precision-fed cecectomized roosters fed the nitrogen-free diet and soybean meal diets at varying crude protein levels^1^.

Item	Dietary CP level (%)								Pooled SEM	*P-*Value
	0	3	6	9	12	15	18	21		
Indispensable amino acids										
Arginine	550.8^b^	588.1^b^	715.7^b^	941.3^b^	1109.6^a^^b^	1080.5^a^^b^	1176.1^a^^b^	1207.9^a^^b^	42.4	<0.001
Histidine	479.9	506.7	634.0	834.9	998.9	1008.0	1081.4	1088.0	68.0	0.093
Isoleucine	309.5^c^	332.9^c^	405.6^b^^c^	530.4^b^	632.0^a^^b^	607.1^a^^b^	660.3^a^^b^	681.8^a^^b^	22.6	<0.001
Leucine	498.4^c^	533.4^c^	654.5^b^^c^	856.9^b^	1019.6^a^^b^	984.4^a^^b^	1069.6^a^^b^	1100.4^a^	36.8	<0.001
Lysine	609.0^c^	654.5^b^^c^	803.6^b^^c^	1051.9^b^	1253.8^a^^b^	1207.9^a^^b^	1308.9^a^^b^	1346.4^a^	47.2	<0.001
Methionine	101.3^b^	109.7^b^	133.3^b^	175.0^a^^b^	208.8^a^^b^	199.1^a^^b^	219.5^a^^b^	222.3^a^	8.6	<0.001
Phenylalanine	435.1^c^	465.2^c^	570.0^b^^c^	746.4^b^	885.0^a^^b^	853.2^a^^b^	926.2^a^^b^	956.9^a^^b^	32.3	<0.001
Threonine	840.8^c^	896.0^c^	1116.8^b^^c^	1453.6^b^	1742.0^a^^b^	1672.2^a^^b^	1801.3^a^^b^	1871.1^a^	65.3	<0.001
Valine	687.6^c^	741.2^b^^c^	900.9^b^^c^	1177.4^b^	1403.8^a^^b^	1348.1^a^^b^	1462.9^a^^b^	1514.8^a^	50.9	<0.001
Dispensable amino acids										
Alanine	634.2^c^	680.1^b^^c^	827.4^b^^c^	1084.3^b^	1283.7^a^^b^	1242.5^a^^b^	1344.8^a^^b^	1393.9^a^	47.4	<0.001
Aspartic acid	1058.0^c^	1134.1^c^	1386.1^b^^c^	1813.3^b^	2160.1^a^^b^	2090.5^a^^b^	2264.3^a^^b^	2338.8^a^	76.8	<0.001
Cystine	604.3^c^	651.4^b^^c^	788.4^b^^c^	1031.4^b^	1228.0^a^^b^	1186.4^a^^b^	1285.1^a^^b^	1331.7^a^	45.3	<0.001
Glutamic acid	1458.0^c^	1558.4^c^	1905.3^b^^c^	2496.2^b^	2962.9^a^^b^	2883.0^a^^b^	3117.0^a^^b^	3220.8^a^	107.4	<0.001
Proline	1194.9^c^	1274.6^b^^c^	1558.2^b^^c^	2039.0^b^	2415.6^a^^b^	2356.1^a^^b^	2532.2^a^^b^	2639.2^a^^b^	91.6	<0.001
Serine	708.4^c^	756.7^c^	938.7^b^^c^	1223.7^b^	1466.6^a^^b^	1410.8^a^^b^	1524.8^a^^b^	1575.5^a^^b^	54.1	<0.001
Total	10210.9^c^	10956.6^b^^c^	13181.2^b^^c^	17341.2^b^	20512.6^a^^b^	20111.6^a^^b^	21900.6^a^^b^	22456.2^a^	757.8	<0.001

CP = crude protein; DMI = dry matter intake.

^1^Values are means with pooled SEM (n = 6). The degrees of freedom between groups are 7 and those within groups are 40. The F-ratios are 7.176, 14.121, 12.692, 15.375, 11.777, 17.333, 17.560, 15.413, 1.910, 15.857, 16.749, 13.967, 14.433, 20.233, 18.504 and 13.196 according to the order of amino acids in the table, respectively.

^a, b, c^Means with the same superscript letters in the row are not significantly different (*P* > 0.05), and those with different letters are significantly different (*P* < 0.05).

**Fig 1:**
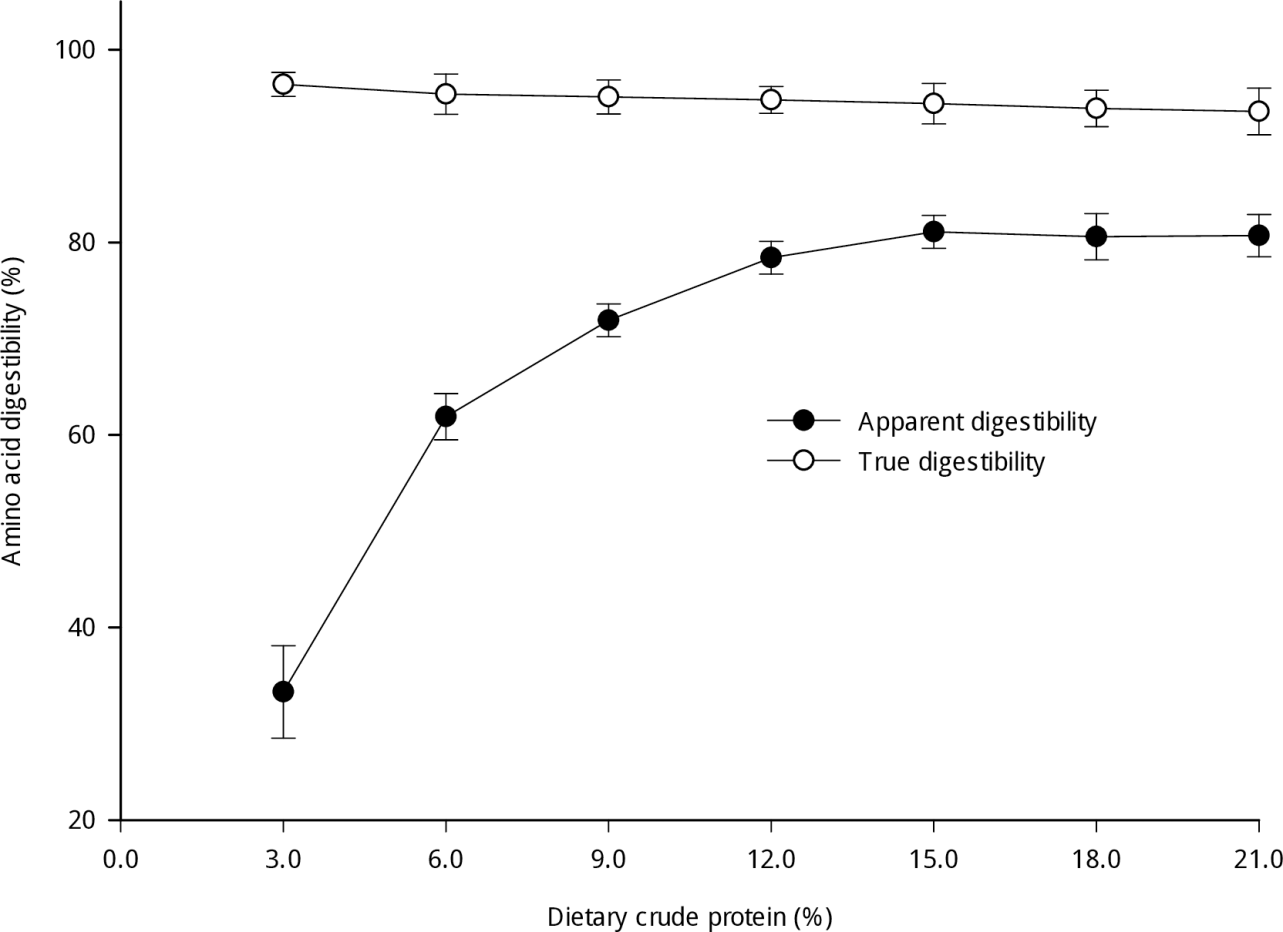
Influence of the dietary crude protein level on the apparent and true digestibility of total AAs in cecectomized roosters fed soybean meal diets. The true digestibility was calculated from the endogenous losses determined by the ^15^N-leucine single-injection method.

The true AA digestibility calculated with the EAALs determined by the ^15^N-leucine single-injection method (total losses) was relatively constant except for a few AAs that had lower digestibility at dietary CP levels of 18% or 21% (Table 6). The apparent digestibility of the total AAs increased nonlinearly with the dietary CP levels, whereas the true digestibility of total AAs was independent of the dietary CP levels (Fig 1).

**Table 6:** True amino acid digestibility calculated from the endogenous losses estimated by the ^15^N-leucine single-injection method in cecectomized roosters fed soybean meal diets at varying crude protein levels^1^.

Item	Dietary CP level (%)							Pooled SEM	*P-*Value
	3	6	6	12	15	18	21		
Indispensable amino acids									
Arginine	97.4	97.2	97.1	97.1	96.9	95.3	95.6	1.78	0.087
Histidine	96.7^a^	95.5^a^^b^	95.6^a^^b^	94.8^a^^b^	95.4^a^^b^	93.1^b^	92.6^b^	1.63	0.005
Isoleucine	97.2	96.9	96.6	96.1	95.7	93.9	94.0	1.25	0.091
Leucine	97.2	97.0	96.7	96.4	96.1	95.5	95.1	0.97	0.081
Lysine	96.8	96.3	95.7	95.5	95.2	93.7	94.5	1.96	0.061
Methionine	98.1	97.0	96.3	96.1	96.8	94.5	94.3	2.02	0.057
Phenylalanine	97.1^a^	96.3^a^^b^	96.4^a^^b^	95.8^a^^b^	95.7^a^^b^	93.2^b^	93.6^b^	1.12	0.002
Threonine	94.9^a^	93.7^a^^b^	93.1^a^^b^	92.4^a^^b^	92.3^a^^b^	90.2^a^^b^	90.0^b^	1.62	0.014
Valine	96.9	94.8	94.5	94.2	94.0	92.8	92.5	2.42	0.121
Dispensable amino acids									
Alanine	96.2	95.2	94.6	94.3	93.7	92.4	92.8	2.25	0.104
Aspartic acid	97.8^a^	96.2^a^^b^	95.6^a^^b^	95.2^a^^b^	94.8^a^^b^	92.7^b^	92.9^b^	1.07	<0.001
Cystine	92.5	89.7	86.6	88.7	86.6	86.9	87.5	2.40	0.074
Glutamic acid	97.2^a^	96.8^a^^b^	96.6^a^^b^	96.4^a^^b^	96.1^b^	94.3^b^	94.7^b^	1.91	0.003
Proline	95.9	93.0	92.4	92.3	92.1	90.3	91.4	1.86	0.152
Serine	96.2^a^	95.5^a^^b^	95.1^a^^b^	94.5^a^^b^	94.2^a^^b^	92.1^b^	92.1^b^	1.19	0.001
Total	96.4	95.4	95.1	94.8	94.4	93.9	93.6	2.19	0.085

CP = crude protein.

^1^Values are means with pooled SEM (n = 6). The degrees of freedom between groups are 6 and within groups are 35. The F-ratios are 3.581, 2.132, 4.958, 3.158, 3.594, 5.351, 5.373, 3.301, 3.816, 4.571, 4.636, 1.694, 4.039, 5.713,4.188 and 3.787 according to the order of amino acids in the table, respectively.

^a, b^Means with same superscript letters in the row are not significantly different (*P*>0.05), and those with different letters are significantly different (*P*<0.05).

## Discussion

One of the aims of the present study was to evaluate the ^15^N-leucine single-injection method within a wide range of dietary CP levels. The results showed that the NL_*cm*_/NL_*p*_ ratios were not influenced by dietary CP levels. Meanwhile, the contributions of endogenous leucine to total leucine in the excreta calculated as NL_*e0*_/NL_*p0*_ and NL_*cm*_/NL_*p*_ were similar at the same dietary CP level. Despite some changes in individual AA proportions (mainly dispensable AAs) when the dietary CP levels were increased from 0 to 21%, the AA profile of total endogenous AAs was relatively constant in the present study. These findings support our assumptions.

The tracer can be given as either a single bolus or continuously when using the isotope labeling technique to trace endogenous AAs [23]. Continuous ^15^N-leucine infusion is the most commonly used technique to determine EAALs in pigs and rats [4–6]. This method assumes that the ^15^N-enrichment of endogenous leucine in excreta is approximately equal to that in plasma (i.e., NL_*e*_ = NL_*p*_) after continuous ^15^N-infusion for 7 to 8 d. Instead of using continuous ^15^N-leucine infusion to obtain a steady state, we proposed the NL_*cm*_/NL_*p*_ ratio remained constant when testing another way of using a single bolus. By regression analysis of the NL_*p*_ value and time in our previous study, we found the optimum time of blood sampling in our method was 23 h after the isotope injection [3]. A single blood sample taken at this time could be used to reflect the weighted mean value of definite integral NL_*p*_ during the sampling period. Additionally, excreta samples from one bird were pooled to obtained the NL_*cm*_ value, which was more representative than the ^15^N-enrichment at a single time point. Therefore, the ^15^N-leucine injection and sample preparation in our method are more economical and simpler than the ^15^N-isotope infusion method.

After a single injection, ^15^N-leucine becomes a part of the pool of the total blood leucine and has the same metabolic rate as unlabeled leucine [3]. Thus, the ratio of labeled to unlabeled endogenous leucine in the excreta has the same tendency to vary as the ratio of labeled to unlabeled leucine in the different sources of endogenous AAs (e.g., plasma, mucosa, or desquamated cells). In other words, the NL_*cm*_/NL_*p*_ ratio indicates the NL_*e*_/NL_*p*_ ratio, which was also confirmed by our present data. Moreover, we proposed a gradient protein method to obtain the AA profiles of total endogenous AAs in birds fed protein-containing diets. The gradient protein method depends on the theory that true AA digestibility is independent of the dietary CP level [2, 14]. Based on several previous studies [24–26], we assumed that variation in the dietary CP levels within a small range had little effect on the EAALs. A study in pigs fed diets with equal graded protein levels of 4% found no significant differences in the EAALs among the adjacent CP levels. In the present study, the EAALs were assumed to be relatively constant or to vary little when the dietary CP levels varied within 3%.

Many studies have generally assumed that the AA profiles of endogenous protein are constant [4, 6, 27]. No significant differences were observed in the ratios of most AAs in the endogenous protein in broiler chickens when increasing the dietary enzyme-hydrolyzed casein concentration from 5% to 20% [24]. This result was almost in agreement with the present data in our study. Although increasing the dietary CP levels stimulates the secretion of digestive enzymes, sloughed cells and mucin [24], the degree of this stimulation source is similar for each secretion. Therefore, the proportion of each excretion source in the total endogenous excretion will not change. Meanwhile, although the relative contribution of each source may vary, the AA composition of a single source tends to be constant [28]. Thus, we infer that the AA profiles of total endogenous AAs are not influenced by dietary CP levels. Moreover, glutamic acid, aspartic acid, threonine, proline and serine dominated the AA profiles of total endogenous AAs, which was consistent with results from chickens [29, 30] and pigs [5, 27]. Additionally, a summary by Boisen et al. [31] indicated that the AA profiles of endogenous protein, which were obtained from different diets and different methods of determination, remained relatively stable. Therefore, the AA profiles of total endogenous AAs in birds fed a nitrogen-free diet were generally acceptable for use in the calculations. Notably, endogenous losses of alanine, cysteine and proline may be underestimated at high dietary CP levels due to the lower proportions of these AAs when the AA profiles of total endogenous AA in the nitrogen-free diet treatment are used.

Another purpose of this study was to estimate total EAALs using the ^15^N-leucine single-injection method and to obtain the true digestibility of AAs in cecectomized roosters fed soybean meal diets with varying CP levels. The results showed that endogenous losses of total AAs increased in response to increases in dietary CP levels, and reached relative equilibrium above 12% dietary CP. Similar changes were found in previous studies [24, 32]. The dietary protein or AA content is one of the main factors affecting EAALs [1]. An increasing dietary CP content likely results in higher specific endogenous losses of AAs, which may be responsible for this result. Moreover, although the apparent AA digestibility nonlinearly increased with the dietary CP level, the true AA digestibility was independent of the respective dietary CP. This finding is in accordance with a well-known conclusion that has been widely demonstrated [2, 15]. This finding also indirectly proves that the ^15^N-leucine single-injection method can be an effective means for determining total EAALs in poultry fed varying levels of protein-containing ingredients.

## Conclusions

The present data support the assumptions of the ^15^N-leucine single-injection method within a wide range of dietary CP levels. The results show that this method allows for the determination the total EAALs and true AAs digestibility in poultry fed protein-containing diets. Notably, endogenous losses of alanine, cysteine and proline may be underestimated at high dietary CP levels. Further studies are also necessary to investigate the accuracy and repeatability of this method, especially using other ingredients as the source of dietary protein.

## Supporting information

S1 Table Analyzed amino acid compositions (g/kg as-fed basis) of the experimental diets. (DOCX)

S2 Table Experimental scheme for the ^15^N-leucine single-injection method, including caecectomy surgery, injection of ^15^N-L-leucine, and sampling times of blood and excreta. (DOCX)

## Abbreviations

EAALs: endogenous amino acid losses
AA: amino acid
CP: crude protein
NL_*e*_: ^15^N-enrichment of endogenous leucine in excreta
NL_*p*_: ^15^N-enrichment of endogenous leucine in deproteinized plasma
NL_*cm*_: ^15^N-enrichment of leucine in crude mucin
APE: atom percent excess
DMI: dry matter intake
AD: apparent digestibility of amino acid
TD: true digestibility of amino acid
