# Supplementary material for: The 15N-leucine single-injection method allows for determining endogenous losses and true digestibility of amino acids in cecectomized roosters
Source: PLoS One. 2017 Nov 22;12(11):e0188525. doi: 10.1371/journal.pone.0188525 (PMC5699825; doi:10.1371/journal.pone.0188525)
Supplement: S1 Table — (DOCX) [file pone.0188525.s001.docx]

**S1 Table** **Analyzed amino acid compositions (g/kg as-fed basis) of the experimental diets**

| Item | Dietary CP level (%) | | | | | | | |
| --- | --- | --- | --- | --- | --- | --- | --- | --- |
|  | 0 | 3 | 6 | 9 | 12 | 15 | 18 | 21 |
| Indispensable amino acids | | | | | | | |  |
| Arginine | 0.10 | 1.93 | 3.74 | 5.55 | 7.28 | 9.16 | 10.97 | 12.77 |
| Histidine | 0.05 | 0.73 | 1.42 | 2.10 | 2.76 | 3.47 | 4.16 | 4.84 |
| Isoleucine | 0.09 | 1.28 | 2.48 | 3.67 | 4.81 | 6.06 | 7.26 | 8.45 |
| Leucine | 0.11 | 2.23 | 4.32 | 6.40 | 8.40 | 10.57 | 12.66 | 14.75 |
| Lysine | 0.10 | 1.69 | 3.28 | 4.86 | 6.37 | 8.02 | 9.60 | 11.18 |
| Methionine | 0.00 | 0.35 | 0.68 | 1.01 | 1.32 | 1.66 | 1.99 | 2.32 |
| Phenylalanine | 0.10 | 1.47 | 2.85 | 4.23 | 5.54 | 6.98 | 8.36 | 9.73 |
| Threonine | 0.07 | 1.09 | 2.10 | 3.11 | 4.09 | 5.14 | 6.16 | 7.16 |
| Valine | 0.04 | 1.37 | 2.64 | 3.91 | 5.13 | 6.46 | 7.74 | 9.01 |
| Dispensable amino acids | | | | | | | |  |
| Alanine | 0.09 | 1.26 | 2.44 | 3.61 | 4.73 | 5.96 | 7.13 | 8.31 |
| Aspartic acid^1^ | 0.21 | 3.21 | 6.20 | 9.19 | 12.07 | 15.19 | 18.19 | 21.18 |
| Cysteine | 0.01 | 0.40 | 0.77 | 1.14 | 1.50 | 1.89 | 2.26 | 2.63 |
| Glutamic acid^1^ | 0.24 | 5.07 | 9.81 | 14.53 | 19.07 | 24.00 | 28.74 | 33.46 |
| Glycine | 0.08 | 1.25 | 2.41 | 3.57 | 4.69 | 5.90 | 7.06 | 8.23 |
| Proline | 0.03 | 1.40 | 2.71 | 4.01 | 5.26 | 6.62 | 7.93 | 9.23 |
| Serine | 0.05 | 1.43 | 2.77 | 4.10 | 5.38 | 6.77 | 8.10 | 9.44 |
| Total | 1.28 | 26.16 | 50.62 | 75.00 | 98.41 | 123.84 | 148.30 | 172.68 |

CP = crude protein; NFD = nitrogen-free diet.

^1^ The respective values for aspartic acid and glutamic acid represent the sum of the concentrations for both the amide and acid derivatives. Tryptophan and tyrosine were destroyed in acid hydrolysis, and were not determined in this study.
