# Supplementary material for: The 15N-leucine single-injection method allows for determining endogenous losses and true digestibility of amino acids in cecectomized roosters
Source: PLoS One. 2017 Nov 22;12(11):e0188525. doi: 10.1371/journal.pone.0188525 (PMC5699825; doi:10.1371/journal.pone.0188525)
Supplement: S2 Table — (DOCX) [file pone.0188525.s002.docx]

**S2 Table** **Experimental scheme for the ^15^N-leucine single-injection method, including caecectomy surgery, injection of ^15^N-L-leucine, and sampling times of blood and excreta**

| Item | ^15^N-L-leucine | Sample | Remarks |
| --- | --- | --- | --- |
| Surgery period |  |  |  |
| Day 0 |  |  | Caecectomy surgery |
| 1 to 48 |  |  | Recovery |
| 49 |  |  | Grouping |
| Experimental periods |  |  |  |
| Period 1^1^ |  |  |  |
| Day 1 to 5 |  |  | Adaption |
| 6 |  |  | Deprivation of diets at 0800 h |
| 7 to 8 |  |  | Precision-feeding at 0800 h on day 7 |
|  |  | Blood | 23 h after precision-feeding |
|  |  | Excreta | 0 to 48 h after precision-feeding |
| Period 2 |  |  | One week of recovery |
| Period 3^2^ |  |  |  |
| Day 1 to 5 |  |  | Adaption |
| 6 |  |  | Deprivation of diets at 0800 h |
| 7 to 8 |  |  | Precision-feeding at 0800 h on day 7 |
|  | ***** |  | ^15^N-leucine injection immediately after precision-feeding |
|  |  | Blood | 23 h after administration |
|  |  | Excreta | 0 to 48 h after precision-feeding |

^1^ Provide samples for determination of basal ^15^N-enrichment of leucine.

^2^ Provide samples for determination of ^15^N-enrichment of leucine after ^15^N-leucine injection.
